# Background Light Suppression for Multispectral Imaging in Surgical Settings

**DOI:** 10.3390/s25010141

**Published:** 2024-12-29

**Authors:** Moritz Gerlich, Andreas Schmid, Thomas Greiner, Stefan Kray

**Affiliations:** Institute of Smart Systems and Services, Pforzheim University, 75175 Pforzheim, Germany; moritz.gerlich@hs-pforzheim.de (M.G.); stefan.kray@hs-pforzheim.de (S.K.)

**Keywords:** multispectral imaging, open surgeries, spectral scanning, background suppression

## Abstract

Multispectral imaging (MSI) enables non-invasive tissue differentiation based on spectral characteristics and has shown great potential as a tool for surgical guidance. However, adapting MSI to open surgeries is challenging. Systems that rely on light sources present in the operating room experience limitations due to frequent lighting changes, which distort the spectral data and require countermeasures such as disruptive recalibrations. On the other hand, MSI systems that rely on dedicated lighting require external light sources, such as surgical lights, to be turned off during open surgery settings. This disrupts the surgical workflow and extends operation times. To this end, we present an approach that addresses these issues by combining active illumination with smart background suppression. By alternately capturing images with and without a modulated light source at a desired wavelength, we isolate the target signal, enabling artifact-free spectral scanning. We demonstrate the performance of our approach using a smart pixel camera, emphasizing its signal-to-noise ratio (SNR) advantage over a conventional high-speed camera. Our results show that accurate reflectance measurements can be achieved in clinical settings with high background illumination. Medical application is demonstrated through the estimation of blood oxygenation, and its suitability for open surgeries is discussed.

## 1. Introduction

Spectral imaging (SI) is a promising technology for providing surgical guidance by enabling image acquisition across multiple wavelength bands simultaneously. These spectrally resolved images allow for the differentiation of biological tissues based on tissue type, malignancy, or perfusion state, as summarized in recent reviews [1,2]. SI can be subdivided into multispectral imaging (MSI) and hyperspectral imaging (HSI), depending on the number of spectral channels. Common to these systems is that raw image data are converted to reflectance measurements, which requires knowledge of the spectral composition of the light source used [3]. However, spectral imaging in dynamically changing and restrictive environments, such as open surgeries, introduces challenges.

In these settings, multiple light sources with different spectral profiles may contribute to the incident light on the tissue, including the surgical light itself, ceiling lights, or head torches worn by the medical staff. Changes in intensity, position, or color temperature will lead to changes in the reflected spectrum, potentially distorting the spectral analysis. Countermeasures such as manual recalibrations throughout the surgery [4] or demanding constant lighting [5] interrupt or restrict the surgical workflow. A method to become independent of such variations has been presented by Ayala et al. [6]. The key idea was to reconstruct the incident spectra through specular reflections. Although successful, this approach relies on the existence of specular reflections and assumes a constant spectral profile over the imaged area. Furthermore, the wavelength range of such systems is restricted to the visible spectrum, as surgical lights typically do not cover the infrared wavelength region [7].

Other SI systems use integrated illumination with a known spectral composition, as demonstrated for endoscopic systems for perfusion monitoring [8,9] and tissue differentiation [10]. However, employing dedicated illumination in open surgeries requires dimming or switching off all other light sources, as shown for multispectral push-broom [11] and spectral scanning systems [12,13], which disrupts the surgical workflow.

We present an alternative approach to address the problem of light variations by combining active illumination with background suppression. In our setup, background light refers to all ambient light sources, including surgical light, while active light denotes a modulated light source at a specific wavelength. Images are then taken in an alternating sequence. Every second image is illuminated by both the background and active light, while every other image captures only the background light. Given a linear camera sensor, the signal of the active light source can be recovered by subtracting the background-only image from the image containing both background and active light. Repeating this process with active light sources at different wavelengths enables spectral scanning and thus multispectral imaging.

This approach must overcome several hurdles. For one, illumination intensities in surgical fields reach values ranging from 40,000 lux to 160,000 lux [14]. If the active illumination is comparably weak, several recordings need to be averaged to achieve a sufficient signal-to-noise ratio (SNR). However, this increases the acquisition time, which, depending on the maximal frame rate of the camera, can introduce motion artifacts as encountered elsewhere [15]. Conversely, increasing the intensity of the active illumination to match surgical light levels presents technical difficulties and could distract medical staff. We address these issues with a novel approach: employing a smart pixel camera with on-chip processing in combination with active illumination. To the best of our knowledge, this is the first work on background suppression in MSI for clinical settings. Our contributions are as follows:

(a) We provide a novel theoretical framework for understanding SNR in spectral scanning under the influence of background light.
(b) For the first time, we evaluate a smart pixel camera for multispectral imaging and compare its performance to a conventional high-speed camera.
(c) We deliver a proof of concept for retrieving accurate reflectance measurements and apply our new method to measure blood oxygenation in vivo.

## 2. Theoretical Background

This section aims to provide an understanding of the primary factors influencing camera performance in background suppression. To this end, a linear camera model as laid out in the EMVA Standard 1288, Release 3.1 [16], is assumed. Although only a single pixel is regarded, the following equations can be expanded for every pixel in the entire image sensor.

The number of photoelectrons collected by a single pixel of a monochrome photosensor is described with Equation (1):

(1) $$\mu_{e}=t_{exp}\cdot A_{eff}\cdot \int_{\lambda_{min}}^{\lambda_{max}}E\left(\lambda \right)\cdot \frac{\lambda }{h\cdot c}\cdot Q\left(\lambda \right)\;d\lambda .$$

Quantities measured in electrons are denoted by subscript *e*, while quantities measured in digital counts are denoted by subscript *c* (see Equation (2)). *E*(*λ*) denotes the spectral irradiance at the photosensor, which is converted to spectral photon flux density by multiplying with $\frac{\lambda }{h\cdot c}$, where λ is the wavelength, *c* is the speed of light, and *h* is Planck’s constant. The quantum efficiency *Q*(*λ*) describes the fraction of photons at a certain wavelength, which are converted into measurable photoelectrons. The exposure time is denoted by *t_exp_*, while the effective pixel area *A_eff_* is the product of the pixel area and the fill factor. Together with the overall system gain *K* and dark current *µ_c,dark_*, the resulting digital signal *µ_c_* can be written as [16]

(2) $$\mu_{c}=\mu_{c,dark}+K\cdot \mu_{e}.$$

A frame pair refers to an image illuminated by the active and background light (*A+B*), from which a second image with only background light (*B*) is subtracted. The resulting signal of a frame pair is $\mu_{c,A}$, as shown in Equation (3):

(3) $$\left(\mu_{c,A+B}+\mu_{c,dark}\right)-\left(\mu_{c,B}+\mu_{c,dark}\right)=\mu_{c,A}.$$

According to [16], the variances of all noise sources are considered to add up linearly and consist of the shot noise ($\sigma_{c,shot}^{2}$), the quantization error ($\sigma_{c,q}^{2}$) as well as noise sources stemming from readout and amplifier circuits ($\sigma_{c,d}^{2}$). The resulting SNR of a frame pair is then given by

(4) $${SNR}_{pair}=\frac{\mu_{c,A}}{\sqrt{2\cdot \sigma_{c,q}^{2}+2\cdot \sigma_{c,d}^{2}+\sigma_{c,A+B,shot}^{2}+\sigma_{c,B,shot}^{2}}}.$$

It should be noted that not only the intensity of the signal $\mu_{c,A}$ but also the shot noise, whose variance grows linearly with the total amount of collected photoelectrons, depends on the exposure time. Nonetheless, the SNR of such a frame pair grows square root-like with exposure time until the sensor is saturated. If the intensity of the active light is relatively weak compared to the background light, multiple frame pairs can be averaged to achieve a target SNR. The total time required for a given target SNR can be determined using the law of averages:

(5) $$t_{required}=t_{pair}\cdot {\left(\frac{{SNR}_{target}}{{SNR}_{pair}}\right)}^{2}.$$

Here, *t_pair_* denotes the time required to record a single frame pair, which might be limited by the overall camera maximum frame rate. The value of $SNR_{pair}$ is determined by measuring the SNR of a frame pair throughout this study. $SNR_{target}$ can be chosen arbitrarily; in this study, a value of 10 is used. The time *t_required_* is therefore camera dependent.

It becomes evident that the suitability of a camera for background suppression depends on the number of photoelectrons a pixel element can collect for a given time frame and by the maximal camera frame rate. Especially environments with high light intensities, which require short exposure times, restrict the benefits from averaging for conventional cameras.

## 3. Materials and Methods

In this section, the cameras used, as well as the light sources, are introduced. Following this, the experimental setup and data processing are described.

### 3.1. High Speed Camera vs. Smart Pixel Camera

The high-speed camera used in this study is a Ximea MQ013RG-ON (Ximea GmbH, Muenster, Germany), hereafter referred to as the Ximea. It employs a global shutter and NIR-enhanced image sensor. The frame rate depends on the spatial resolution and bit depth. In this study, the bit depth was set to 10 bits, while the spatial resolution was chosen to be 640 × 512 pixels, allowing for a frame rate of 773 Hz. To perform background suppression, images must be taken such that every second image is additionally illuminated with the desired light source. These images are then individually transferred to the computer and digitally subtracted to form frame pairs, as described by Equation (3).

The smart pixel camera, referred to as C4, is a dual-phase lock-in camera (C4.0S41U, Heliotis AG, Root, Switzerland). It features an image sensor with a specialized pixel architecture with a spatial resolution of 512 × 542 pixels, which measures the in-phase (I) and quadrature (Q) components of a signal at a given reference frequency, $f_{ref}$.

**Figure 1:**
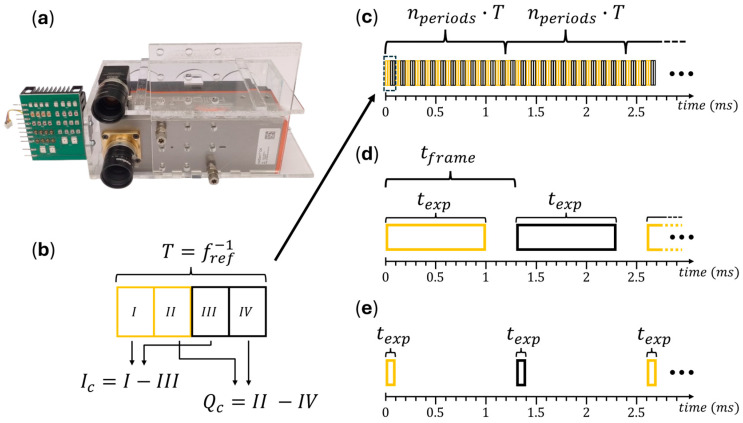
(**a**) Ximea camera (top), Heliotis C4 camera (bottom), and multispectral LED array (left); (**b**) single demodulation period of the C4 camera, subdivided into four quarter periods. Active illumination is switched on during quarter periods highlighted in orange and switched off in quarter periods depicted as black. The resulting I- and Q-components are summed internally over *n_periods_* to form the in-phase and quadrature images; (**c**) operation principle of the C4 with *T* = 0.1 ms and *n_periods_* = 12. Each sequence of duration *n_periods_*·*T* results in one I/Q image, which is stored digitally on the camera’s internal memory. The resulting images are transferred after one burst; (**d**) background suppression as realized with the Ximea for an exposure time of 1 ms and maximal frame rate of 773 fps; (**e**) same as (**c**) with an exposure time of 100 µs. Note the dead times between frames due to the limited frame rate.

The working principle of the C4 involves dividing each demodulation period by duration $T=1/f_{ref}$ into four quarter-periods (I, II, III, and IV) as shown in Figure 1b. The I-component of one such period is calculated by taking the difference between the signal in quarter-periods I and III, while the Q-component is obtained from the difference between quarter-periods II and IV. This process is repeated for a set number of demodulation periods, $n_{periods}$. The resulting in-phase and quadrature images are formed by summing the respective I- and Q-components over $n_{periods}$. This principle is realized directly on the sensor in an analog manner, and only the resulting in-phase and quadrature images are transmitted after a burst.

As with the Ximea, the C4 is synchronized with a rectangularly modulated light source operating at a constant reference frequency. This allows for summing up the in-phase and quadrature images directly, which in turn facilitates the comparison of both cameras. The time for such a background-suppressed frame, which is conceptually equivalent to a frame pair, is then given by

(6) $$t_{pair}=n_{periods}\cdot f_{ref}^{-1}.$$

Both cameras were equipped with the same 16 mm fixed focal length lens (M1628-MPW3, Computar, Singapore, Singapore).

### 3.2. Light Sources

**Figure 2:**
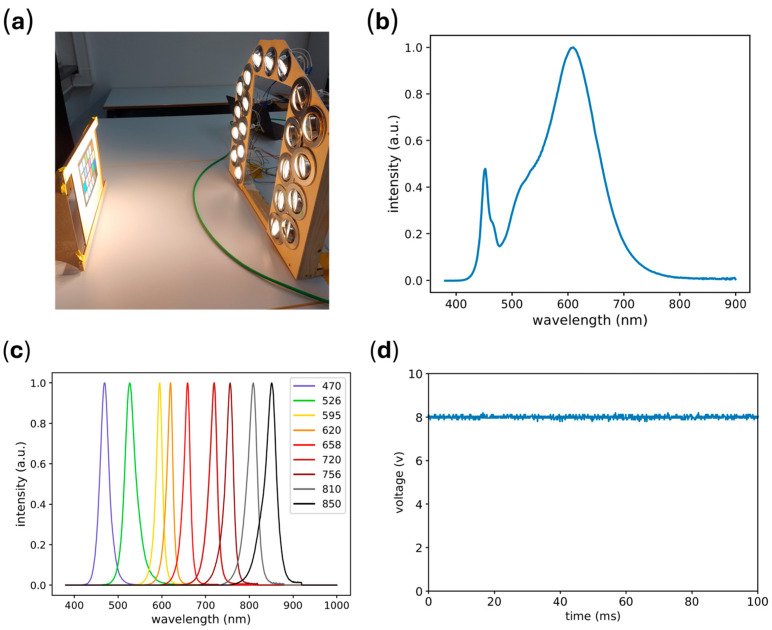
(**a**) Setup at 56,000 lux background illumination; (**b**) spectral composition of the LED spotlights (background light); (**c**) spectral composition of the multispectral LED array; (**d**) temporal intensity of LED spotlights as measured with the photoreceiver.

The actively modulated illumination is provided by 36 high-power SMD-LEDs, as shown in Figure 1a. Four identical LEDs contribute to each spectral band, resulting in nine different bands spanning the visible (VIS) to NIR spectral range, as displayed in Figure 2b. The LEDs are powered by a 250 MHz driver (iC-HG30 EVAL HG1D, iC-Haus GmbH, Bodenheim, Germany). The intensities of the LEDs are controlled via potentiometers situated on the driver. The synchronization of the active illumination is achieved by connecting the driver to a microcontroller board (Uno R3, Kuman, Shenzhen, China), which in turn receives the synchronization signals from both cameras. The background light (see Figure 2a), which mimics a surgical light source, is provided by 21 LED spotlights driven by a laboratory power supply. The spectral composition of the background light is shown in Figure 2b. The intensity can be adjusted by varying the current at the power supply to meet the desired illumination, as measured with a luxmeter (PeakTech 5165, PeakTech Prüf- und Messtechnik GmbH, Ahrensburg, Germany). The background light (Figure 2d) is flicker-free, as verified using a photoreceiver (2031 Large Area Photoreceiver, Newport Corporation, Irvine, CA, USA). Modern LED-based surgical lights are often designed to provide consistent, flicker-free illumination. Therefore, we assume that our light source is comparable to surgical lighting systems.

### 3.3. Experimental Setup and Data Processing

#### 3.3.1. Camera Settings

Both cameras were set to a bit depth of 10 bits. Care was taken to ensure the image sensor was not saturated, limiting the exposure time. Conversely, the standard deviation of the shot noise increases as a square root function with increasing exposure time while the signal increases linearly. Therefore, the optimal exposure time maximizes the signal strength while avoiding saturation. In this sense, the exposure time of the Ximea was adjusted once for each measurement series to result in a digital count of 900 while imaging the white target under background illumination. Throughout all experiments, the Ximea reached its maximal frame rate of 773 fps. Therefore, a frame pair could be recorded every 2.59 ms. As with the Ximea, the C4 needed to avoid saturation by the background light. The demodulation frequency was set to 10 kHz, ensuring that each quarter period spanned 25 µs, sufficient to avoid sensor saturation in all measurements.

#### 3.3.2. SNR Test

**Figure 3:**
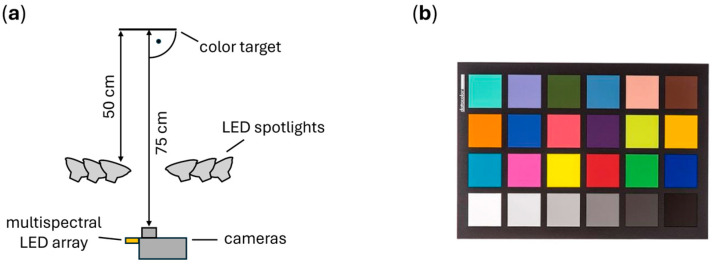
(**a**) Schematic depiction of the experimental setup. The distance of the cameras to the imaged object was fixed at 75 cm throughout this work; the distance from the LED spotlights to the target was set to be 50 cm; (**b**) color target. The white patch at the bottom left was used as the test object.

The focus of the first experiment was to determine the required time to capture a test object with a desired signal-to-noise ratio for a single color under background illumination. Both cameras were positioned 75 cm away from a color target (Spyder Checkr 24, Datacolor GmbH, Marl, Germany), as depicted in Figure 3a. The cameras were adjusted such that the white patch of the color target, serving as the test object, was imaged at the center of the camera sensors. The background illuminance on the test object was measured with a luxmeter and adjusted to 28,000 lux; 56,000 lux; and 96,000 lux, respectively, using the 21 LED spotlights positioned at a distance of 50 cm to achieve these high illuminance levels.

The active illumination was provided by the LEDs with a central wavelength of 620 nm for this test. For each of the three background light levels, seven different levels of active light intensity were used, resulting in 21 combinations of active and background light. A total of 3000 frame pairs were recorded by each camera for every combination. The SNR of a single frame pair was calculated by averaging the pixel-wise SNR as measured over the target area.

In this study, the target SNR was set to 10, a common benchmark for comparing images in computer vision. The required time to achieve the target SNR was calculated according to Equation (5). Since the target SNR is constant, this time is determined by the SNR of a single frame pair, which is a measured quantity. Therefore, the individual camera parameters, such as exposure time, are irrelevant as long as the image sensor is not saturated. To avoid overexposure, $t_{exp}$ of the Ximea camera was chosen to match a signal level of 900 digital counts for each of the three background levels. However, this results in very short exposure times compared to the time for one frame pair of 2.59 ms, limited by the maximum frame rate. The C4 camera was set to $t_{pair}=0.4\;ms$, avoiding overexposure for all background light settings. The camera settings used throughout the SNR test are summarized in Table 1.

#### 3.3.3. Spectral Validation

The second experiment was a direct feasibility test of the proposed background suppression scheme. Reflectance measurements were performed with both cameras under background illumination and compared against ground truth reflectance measurements.

First, the ground-truth reflectance of individual tiles of the color target was measured with a spectrometer (LR 2 broadband spectrometer, Lasertack GmbH, Fuldabrück, Germany). The color target and a white reflectance target were both illuminated using a broadband halogen light source, covering the VIS and NIR range. Reflectance values were then calculated via

(7) $$R_{spect}\left(\lambda \right)=\frac{I_{raw,spect}\left(\lambda \right)-I_{d,spect}\left(\lambda \right)}{I_{w,spect}\left(\lambda \right)-I_{d,spect}\left(\lambda \right)}.$$

$R_{spect}\left(\lambda \right)$ denotes the reflectance of the respective tile, as measured with the spectrometer, $I_{raw,spect}\left(\lambda \right)$ denotes the raw signal as reflected from the tile, $I_{w,spect}\left(\lambda \right)$ denotes the raw signal as reflected from the (white) reference material, and $I_{d,spect}\left(\lambda \right)$ represents the dark current.

Reflectance images were then recorded by both cameras in an analogous manner. First, the background light was set to illuminate a larger area with approximately 56,000 lux, and camera parameters were tuned to measure a full multispectral cube with 9 colors in ~100 ms. Second, both cameras recorded multispectral cubes of the white reflectance target (DIN A4 size), which covered the same area as the color target. Third, the color target was set as the current scene, and multispectral cubes were collected.

Since the active illumination from the LEDs light the scene in a non-uniform manner caused by the spatial arrangement of individual LEDs and the Lambertian emitter profile, the raw signal reflected from the white reference material must be accounted for on a pixel-wise level. The reflectance spectra at a given pixel position were therefore calculated via Equation (8):

(8) $$R_{cam}\left(n,x,y\right)=\frac{I_{raw,cam}\left(n,x,y\right)}{I_{w,cam}\left(n,x,y\right)}.$$

Here, *n* denotes the number of the spectral channel, $R_{cam}\left(n,x,y\right)$ denotes the reflectance measured with the camera, $I_{raw,cam}\left(n,x,y\right)$ the raw signal of the measured object and $I_{w,cam}\left(n,x,y\right)$ the raw signal as reflected from the white reference material at a given pixel position (x,y). The dark current cancels out due to the background suppression scheme and does not need to be considered here.

Similarly to a filter-wheel-based MSI system [15], the reflectance recorded by the cameras should approximate the ground-truth reflectance of the tiles, weighted by the spectral profile of the respective LEDs as shown in Equation (9):

(9) $$R_{spect,weighted}\left(n\right)=\int_{\lambda_{min,n}}^{\lambda_{max,n}}{S\left(n,\lambda \right)\cdot R}_{spect}\left(\lambda \right)d\lambda .$$

Here, $S\left(n,\lambda \right)$ denotes the spectral distribution of the LEDs of channel *n*, normalized to an area of 1. Pixel-wise reflectance values recorded by the cameras were then compared to these weighted spectrometer values via the spectral angle mapper (SAM) [17]:

(10) $$SAM(x,y)={cos}^{-1}\left(\frac{\sum_{n=1}^{9}R_{cam}\left(n,x,y\right)\cdot R_{spect,weighted}(n)}{\sqrt{\sum_{n=1}^{9}R_{cam}^{2}\left(n,x,y\right)}\cdot \sqrt{\sum_{n=1}^{9}R_{spect,weighted}^{2}\left(n\right)}}\right).$$

Both cameras were set to match a recording time of approximately 100 ms per multispectral cube, as summarized in Table 2. For the Ximea, four frame pairs per spectral channel were averaged. As a frame pair is recorded with $t_{pair}=2.59\;ms$, this results in a value of $t_{color}$ = 10.36 ms per spectral channel and $t_{cube}=93.24$ ms. The C4 camera, operating at 10 kHz, allowed the number of demodulation periods to be set in increments of two, with a minimum of four and a maximum of 100 periods. Therefore, the number of demodulation periods was set to 52, with two such frames contributing to each spectral channel. Thereby each spectral channel was recorded over 10.4 ms, while the entire multispectral cube was recorded in 93.6 ms. The resulting mismatch in total recording time between both cameras is minimal and does not significantly impact the comparison. 

The LEDs operated near their maximal intensity throughout the spectral validation and perfusion assessment. The combined illuminance of each LED group, consisting of four identical LEDs each, was measured at the center of the white target using the luxmeter. The LEDs were rectangularly modulated in intensity with a frequency of 10 kHz and a duty cycle of 50%. The measured illuminances are shown in Table 3.

#### 3.3.4. Perfusion Assessment

The third experiment aimed to evaluate whether either camera can realistically be used to perform perfusion assessment in a medical setting. To this end, an occlusion test was performed while data were recorded with our optical system. The participant was seated on a chair with an inflatable cuff (S83, Speidel+Keller GmbH, Jungingen, Germany) placed around the right forearm. The distance from the cameras to the scene was set to 75 cm. The background light was set to illuminate a larger area with approximately 56,000 lux. Both cameras recorded multispectral cubes of the right hand of the participant, successively every two seconds. The time for acquiring a multispectral cube was again set to ~100 ms for both cameras. After the first 40 s, the occlusion pressure was adjusted to 220 mmHg to create an arterial occlusion. After two minutes, the cuff was deflated to allow reperfusion while the data recording continued for another minute. As in the spectral validation experiment, the multispectral cubes were normalized to reflectance measurements on a pixel-by-pixel level using the same white reflectance target of DIN A4 size.

A pixel-wise estimation of oxygen saturation (SO_2_) was calculated based on the modified Beer-Lambert law, as employed in works by Clancy et al. [15,18]. This model assumes that (i) blood is the dominant absorber within the imaged tissue, (ii) the path length of light is identical for each wavelength, and (iii) scattering losses are constant over all measured wavelengths. Considering that oxygenated and deoxygenated blood absorbs light differently depending on the wavelength, it is possible to estimate the relative concentrations of these chromophores based on reflectance measurements. To this end, pixel-wise reflectance measurements were first converted to absorbance:

(11) $$A\left(\lambda ,x,y\right)=-ln\left(R\left(\lambda ,x,y\right)\right).$$

Secondly, the concentrations of oxyhemoglobin and deoxyhemoglobin, denoted as $\left[HbO_{2}\right]$ and $\left[Hb\right]$, respectively, were calculated by fitting the recorded absorbance to known extinction coefficients of the respective hemoglobin species denoted as $\epsilon \left(\lambda \right)$ [19]:

(12) $$A\left(\lambda ,x,y\right)=\left[HbO_{2}\left(x,y\right)\right]\epsilon_{HbO_{2}}\left(\lambda \right)+\left[Hb\left(x,y\right)\right]\epsilon_{Hb}\left(\lambda \right)+\alpha \left(x,y\right).$$

Here, α is a constant accounting for losses due to scattering and other chromophores. Lastly, the oxygen saturation is calculated via these fitted values:

(13) $${SO}_{2}\left(x,y\right)=\frac{\left[HbO_{2}\left(x,y\right)\right]}{\left[HbO_{2}\left(x,y\right)\right]+\left[Hb\left(x,y\right)\right]}.$$

This process was repeated for each pixel (x,y) of the imaged tissue. Although two wavelengths are sufficient for this analysis [20,21], we used four channels with central wavelengths of 658 nm, 720 nm, 756 nm and 810 nm to optimize for spectral sensitivity of the camera, thereby improving stability. The SO_2_ value was then encoded as a color overlay to the image.

## 4. Results

### 4.1. SNR Test

**Figure 4:**
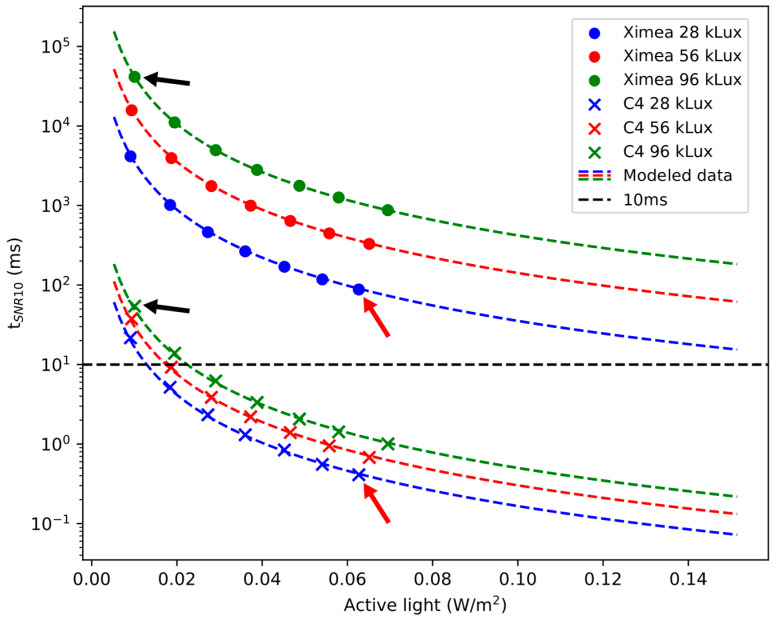
Required time to record the test object with a target SNR of 10 for one spectral channel. The intensity of the active light is depicted on the *x*-axis. The ratio of active illumination to background light ranged from 2.4% (red arrow) down to 0.1% (black arrow).

Throughout the SNR test, as detailed in Section 3.3.2., images of a test object were acquired while the intensities of the background and active illumination were varied. The results for recorded data (solid dots) and modeled data according to Chapter 2 (dashed lines) are shown in Figure 4.

The irradiance of both the active illumination and background light on the camera sensors was measured accordingly. The required time to record the actively illuminated test object with a target SNR of 10 depends on the SNR of a single frame pair. All measured values of the Ximea are above 80 ms, while the required time for the C4 was mostly measured to be below 10 ms. The modeled data show that the Ximea does not reach values below 10 ms for a target SNR of 10.

### 4.2. Spectral Validation

A total of 100 multispectral reflectance images of a color target were acquired by each camera, as described in Section 3.3.3. No image smoothing was performed, and all reflectance spectra were taken on a pixel-wise level.

**Figure 5:**
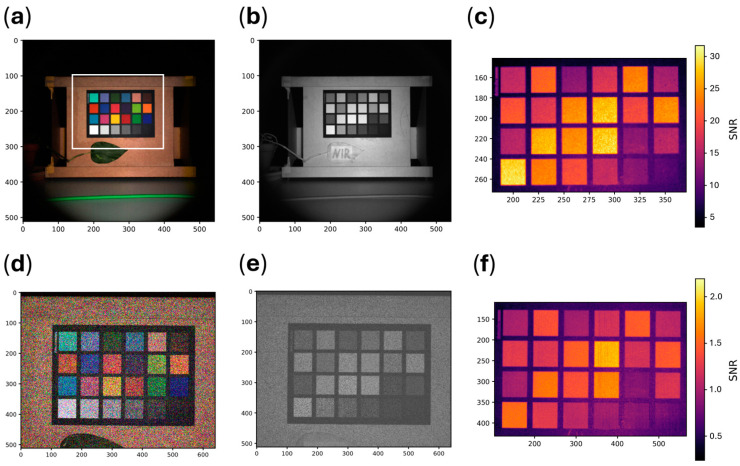
(**a**) C4 reconstructed RGB image. The highlighted box indicates the field of view of the Ximea camera; (**b**) C4, single-channel image with a central wavelength of 810 nm; (**c**) SNR as recorded with the C4, averaged over all channels. The SNR image was cropped to the size of the color target; (**d**) Ximea, reconstructed RGB image; (**e**) Ximea, single-channel image with a central wavelength of 810 nm; (**f**) SNR as recorded with the Ximea, averaged over all channels. The SNR image was cropped to the size of the color target; (**a**–**f**) values on the image axes denote the pixel position.

**Figure 6:**
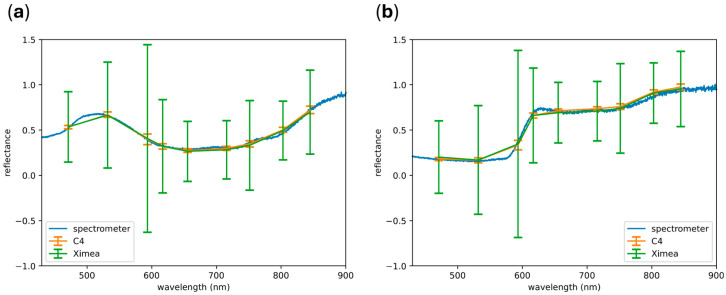
Exemplary reflectance spectra of the color target for the dark-blue tile (**a**) and the salmon tile (**b**). Vertical bars indicate the standard deviation of pixel-wise recorded reflectance spectra. Statistics were estimated from 1000 pixel-wise reflectance spectra for each tile of the color target.

Reconstructed RGB images derived from single multispectral cubes are shown in Figure 5a,d. Due to differences in sensor size, the field of view varies between the two cameras, as both were equipped with the same lens. The RGB images reconstructed by the C4 exhibit little discernible noise, in contrast to those reconstructed by the Ximea, which show a high level of noise. These differences are also evident in the single spectral channel images, as shown in Figure 5b,e. The pixel-wise SNR, averaged across all channels, is displayed in Figure 5c,f. On average, the C4 achieved a pixel-wise SNR of around 20, while the Ximea showed an average pixel-wise SNR of around 1, as summarized in Table 4. When averaging the reflectance spectra across multiple pixels for each tile, both cameras closely matched the ground truth, as shown in Figure 6. However, when examining individual spectra, the Ximea exhibited a high standard deviation across all spectral bands compared to the C4.

The average SAM score between the ground truth data and the pixel-wise reflectance measurements is shown in Table 4. A generally low SAM score, and thereby high similarity, could be seen for the C4, while the Ximea showed a low similarity.

### 4.3. Perfusion Assessment

**Figure 7:**
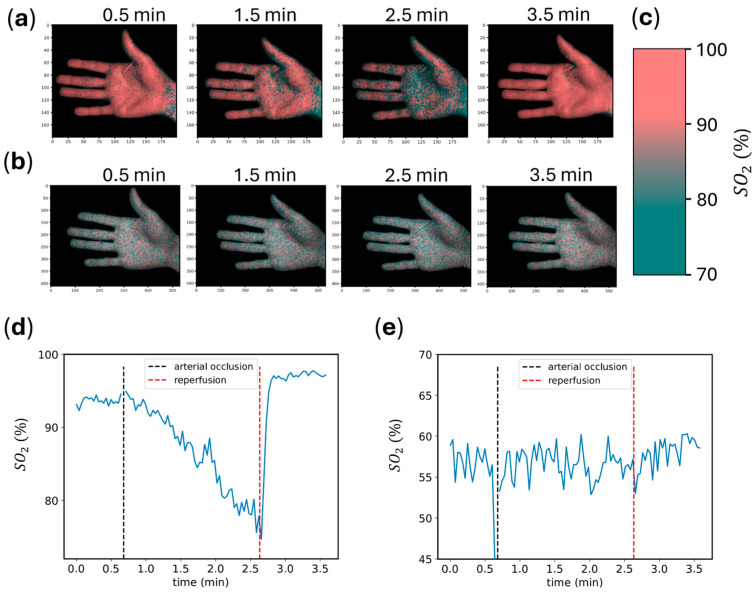
Perfusion assessment throughout the arterial occlusion: (**a**) false color images as recorded by the C4. Images were cropped for better comparison. Values on the image axes denote the pixel position; (**b**) false color images as recorded by the Ximea. Images were cropped, and gray-level images were color-coded for enhanced visibility. Values on the image axes denote the pixel position; (**c**) color coding for the C4 as a function of tissue oxygenation. The color coding was centered at 85% SO_2_ for the C4 and at 57% SO_2_ for the Ximea, according to (**d**,**e**); (**d**) average pixel-wise oxygen saturation over time as recorded by the C4; (**e**) average pixel-wise oxygen saturation over time as recorded by the Ximea.

Multispectral reflectance images were recorded throughout an occlusion test, as described in Section 3.3.4. A pixel-wise estimation of tissue oxygenation based on known reference spectra was performed. A qualitative assessment is shown in Figure 7a–c, where a clear difference in tissue oxygenation before, during, and after the arterial occlusion can be seen. Figure 7d,e show the average pixel-wise estimate of tissue oxygenation over time. A gradual decline can be seen after the creation of the arterial occlusion, with a sharp increase following the deflation of the cuff. A change in tissue oxygenation, both qualitatively and quantitatively, was only discernible for the C4.

## 5. Discussion

We examined whether a combination of spectral scanning and background suppression can realistically enable multispectral imaging during open surgeries. To this end, a series of experiments was performed under lighting conditions close to those encountered in a clinical setting. A framerate-limited, sequential camera was compared to an on-chip smart pixel camera, which realizes background suppression directly on the sensor.

Intuitively, one can assume that the high illumination from the surgical light leads to short exposure times, as sensor saturation must be avoided. Indeed, for the sequential camera, exposure times below 100 µs became necessary, starting at 28,000 lux within the SNR test. At 773 fps, more than 90% of the time, no signal was collected. In contrast, the on-chip smart pixel camera generally acquires images without intermediate data transfer and thereby collects all the available signals. This effect became more pronounced with increasing background illumination, as can be seen in Figure 4 for the measurement series at 28,000 lux and 56,000 lux. Doubling of the background illumination required halving the exposure time of the sequential camera. Consequently, only half as many photoelectrons from the active light were collected. This halved the SNR of a frame pair, as the signal level halves while still collecting ~900 digital counts mainly from the background light. This, in turn, quadruples the required time of the Ximea to achieve the target SNR. Conversely, for the on-chip smart pixel camera, doubling the background light intensity increased the shot noise by a factor of $\sqrt{2}$, which, considering that the shot noise is the main source of noise, led only to a doubling of the required time. This frame rate-dependent trend continued further from 56,000 lux to 96,000 lux. Even in the best case under relatively low background illumination, the sequential camera required more than 80 ms for a target SNR of 10. Expanding this to 9 spectral channels would result in recording times of at least 720 ms for one multispectral cube, making it impractical for real-world use due to motion artifacts.

The discrepancy in camera performance can further be attributed to differences in sensor size and pixel area. The effective pixel area of the on-chip smart pixel camera is 316.8 µm^2^ according to the datasheet, while the effective pixel area accounting for the fill factor of the Ximea was experimentally estimated to be 19.3 µm^2^. This difference in effective pixel area by itself leads to an SNR difference in factor ~4 if variations in quantum efficiency and the spectral composition of light sources are disregarded. However, at clinical background illumination levels, framerate becomes the dominant factor in camera performance. At 56,000 lux, the SNR difference between the two cameras was approximately a factor of 20, primarily driven by the C4’s ability to fill nearly 100% of the exposure time with light from active illumination.

In the spectral validation experiment, both cameras were generally capable of providing accurate reflectance measurements by averaging many frames. If time constraints were introduced to mimic motion artifacts as present in surgical settings, the low SNR as recorded with the sequential camera rendered pixel-wise reflectance measurements infeasible. Conversely, the on-chip smart pixel camera provided a high signal-to-noise ratio for pixel-wise reflectance measurements. This trend was also visible in the direct comparison of the RGB images of both cameras under high background illumination.

Similarly, the assessment of tissue oxygenation, which generally relies on correct reflectance measurements, was demonstrated for the on-chip smart pixel camera, while no meaningful perfusion assessment could be performed with the sequential camera. The temporal progression of tissue oxygenation, including the overshoot after reperfusion, matched that reported in the literature [22,23]. A limitation of this experiment was the absence of a reference system with which the absolute oxygenation measurements could be validated. However, this would require an invasive procedure for taking blood samples, which is out of the scope of this work.

The experiments on spectral validation and perfusion assessment required the background light to cover the entire color target or the participant’s hand, in contrast to the SNR test, where the background illumination was concentrated on a small white patch of the color target. As a result, the achievable illuminance over the imaged area was limited to 56,000 lux.

This raises the question of whether the results obtained in these experiments can be scaled to operating room conditions. The distance of the active light to the target (75 cm) represents a realistic value for clinical settings and exceeds the 50 cm distance commonly used by the TIVITA^®^ system (Diaspective Vision GmbH, Am-Salzhaff-Pepelow, Germany) [11]. However, surgical luminaires can provide illuminance levels exceeding 100,000 lux. As demonstrated in the SNR test for the C4 camera, an increase in background illumination by a given factor leads to an increase in shot noise proportional to the square root of that factor. To maintain the same SNR under higher background light levels at identical recording times, the active illumination must also be scaled proportionally to the square root of that factor. For example, if the background illumination increases by a factor of 3, from 56,000 lux to above 150,000 lux, the active illumination must increase by approximately 1.7 times. In this setup, this would require approximately 7 LEDs per spectral band to be used instead of the original 4. The illuminance of the active light, as tabulated in Table 3, would scale accordingly. Given our setup, the brightest color channel, that is, the green channel, would reach lux values of ~ 460 lux in the above example. Since the channels are measured sequentially, the active illumination would be below the recommended value of 1000 lux for ambient lighting [14].

Overall, to implement the proposed background suppression in a clinical setting, a conventional camera is not sufficient, as frame rates of several thousand fps are required to achieve a similar performance compared to the smart pixel camera. The applicability of the C4 was demonstrated for this use case.

Currently, the LEDs used for this study were selected for achieving a wide coverage of the VIS and NIR region, which is not optimal for medical tasks such as determining blood oxygenation levels. However, the spectral ranges of these LEDs can be matched to the intended use case [24].

Currently, all captured images must be read out together after each burst of the C4 camera. This restricts the time for acquiring consecutive multispectral cubes, as only a limited amount multispectral cubes fit into the memory of the camera. For example, the settings for the spectral validation and perfusion assessment led to readout times of 265 ms for one multispectral cube. This, together with the integration time of 93.6 ms, limits the maximal rate at which multispectral cubes are recorded to ~2.8 Hz. This low multispectral frame rate can be overcome by parallel acquisition during readout, which is currently not feasible due to firmware restrictions. We estimate that multispectral frame rates of 10 fps will be feasible with improved firmware.

## 6. Conclusions

In this work, we demonstrated a novel method to become independent of background illumination in multispectral imaging. Our system relies on a modulated light source and an on-chip smart pixel camera. Even at high background illumination, it was possible to ensure accurate reflectance measurements and record dynamic biological processes without considering the spectral contribution from other light sources. Therefore, we are confident that our approach will become a valuable tool for multispectral imaging in clinical settings. In future work, we aim to assess our system for the task of perfusion assessment during open surgical procedures involving bowel anastomosis.

## Figures and Tables

**Table 1:** Camera settings for the three-measurement series of the SNR test. *t_color_* denotes the time over which a frame pair—or, in the case of the C4, a background-suppressed frame—is recorded.

	Ximea		C4
Illuminance [lux]	*t_exp_* [µS]	*t_color_* [ms]	*t_color_* [ms]
28,000	100	2.59	0.4
56,000	50	2.59	0.4
96,000	30	2.59	0.4

**Table 2:** Camera settings throughout the spectral validation and perfusion assessment. *t_color_* denotes the recording time for each spectral channel, while *t_cube_* denotes the total recording time of one multispectral cube.

		Ximea			C4	
Experiment	Illuminance [lux]	*t_exp_* [µS]	*t_color_* [ms]	*t_cube_* [ms]	*t_color_* [ms]	*t_cube_* [ms]
Spectral Validation	56,000	50	10.36	93.24	10.4	93.6
Perfusion Assessment	56,000	50	10.36	93.24	10.4	93.6

**Table 3:** Intensity of the active illumination, as measured with the luxmeter. Each LED group was measured at the center of the white target. The NIR channels are not visible to the human eye, resulting in lux values of zero. However, their intensities are comparable to those of the other color channels.

Central Wavelength [nm]	Illuminance [lux]
470	53
526	273
595	151
620	227
658	112
720	5
756	0
810	0
850	0

**Table 4:** Average SAM score between individual spectra and ground truth data and average SNR over all channels and tiles of the color target. Low SAM scores indicate high spectral similarity.

Camera	SAM α	SNR
Ximea	0.730 ± 0.250	1.26
C4	0.061 ± 0.029	20.46

## Data Availability

The data presented in this study are available upon request from the first author.
